# Targeting the *Plasmodium falciparum*’s Thymidylate Monophosphate Kinase for the Identification of Novel Antimalarial Natural Compounds

**DOI:** 10.3389/fcimb.2022.868529

**Published:** 2022-05-25

**Authors:** Kweku S. Enninful, Samuel K. Kwofie, Mark Tetteh-Tsifoanya, Amanda N. L. Lamptey, Georgina Djameh, Samuel Nyarko, Anita Ghansah, Michael D. Wilson

**Affiliations:** ^1^ Department of Parasitology, Noguchi Memorial Institute for Medical Research, University of Ghana, Accra, Ghana; ^2^ Department of Biomedical Engineering, School of Engineering Sciences, University of Ghana, Accra, Ghana; ^3^ West African Centre for Cell Biology of Infectious Pathogens, College of Basic and Applied Sciences, University of Ghana, Accra, Ghana; ^4^ Stritch School of Medicine, Loyola University of Chicago, Maywood, IL, United States

**Keywords:** *Plasmodium falciparum*, aurantiamide acetate, *Pf*TMPK, *Artemisia annua*, natural compounds

## Abstract

Recent reports of resistance to artemisinin-based combination drugs necessitate the need to discover novel antimalarial compounds. The present study was aimed at identifying novel antimalarial compounds from natural product libraries using computational methods. *Plasmodium falciparum* is highly dependent on the pyrimidine biosynthetic pathway, a *de novo* pathway responsible for the production of pyrimidines, and the parasite lacks the pyrimidine salvage enzymes. The *P. falciparum* thymidylate monophosphate kinase (*Pf*TMPK) is an important protein necessary for rapid DNA replication; however, due to its broad substrate specificity, the protein is distinguished from its homologs, making it a suitable drug target. Compounds from AfroDB, a database of natural products originating from Africa, were screened virtually against *Pf*TMPK after filtering the compounds for absorption, distribution, metabolism, excretion, and toxicity (ADMET)-acceptable compounds with FAF-Drugs4. Thirteen hits with lower binding energies than thymidine monophosphate were selected after docking. Among the thirteen compounds, ZINC13374323 and ZINC13365918 with binding energies of −9.4 and −8.9 kcal/mol, respectively, were selected as plausible lead compounds because they exhibited structural properties that ensure proper binding at the active site and inhibitory effect against *Pf*TMPK. ZINC13374323 (also called aurantiamide acetate) is known to exhibit anti-inflammatory and antiviral activities, and ZINC13365918 exhibits antileishmanial activity. Furthermore, aurantiamide acetate, which is commercially available, is a constituent of *Artemisia annua*, the herb from which artemisinin was derived. The compound also shares interactions with several residues with a potent thymidine analog inhibitor of *Pf*TMPK. The anti-plasmodial activity of aurantiamide acetate was evaluated *in vitro*, and the mean half-maximal inhibitory concentration (IC_50_) was 69.33 μM when synchronized *P. falciparum* 3D7 culture was used as compared to IC_50_ > 100 μM with asynchronized culture. The significance of our findings within the context of malaria treatment strategies and challenges is discussed.

## Introduction

*Plasmodium falciparum* is among the five *Plasmodium* parasites that cause human malaria and is also responsible for the most severe form of the disease (Tuteja, 2007; Ludin et al., 2012; Spitzmüller and Mestres, 2013; Bhatt et al., 2015). Recent studies have reported *P. falciparum* to be developing resistance to current major antimalarial drugs (Egwu et al., 2022), which warrants the identification and development of new antimalarials as a necessity. Throughout the history of malaria chemotherapy, the most successful antimalarials have been natural products. Antimalarials such as chloroquine were developed from quinine, which was extracted from the bark of the *Cinchona* tree from South America. Artemisinin was also obtained from *Artemisia annua* originating from China (Tajuddeen and Van Heerden, 2019). Medicinal herbs have generally proven to be very effective drugs against parasitic diseases (Siddiqui et al., 2014). Even though both chloroquine and artemisinin have been plagued with resistance to the parasite, research into natural product antimalarials remains a priority. Out of about 1,524 compounds with anti-plasmodial activity reported between 2010 and 2017, 39% were natural products, with 29% of the compounds having half-maximal inhibitory concentration (IC_50_) ≤ 3.0 µM against at least one *Plasmodium* strain (Tajuddeen and Van Heerden, 2019). This reinforces the urgent need to exploit natural products to unravel future potent biotherapeutic molecules.

Research efforts at developing new antimalarials have uncovered diverse pathways and protein targets of which some are novel (Belete, 2020; Agamah et al., 2021). It is worthwhile to mention that new antimalarials, currently in the clinical trial phase, have been identified against critical pathways and targets (Kumar et al., 2018; Belete, 2020). However, a good number remain unexploited, while others have not yielded any potent drugs. Identification of new antimalarial targets from novel pathways not associated with resistance must be explored by screening diverse compound libraries (Ludin et al., 2012; Spitzmüller and Mestres, 2013).

Thymidylate monophosphate kinase catalyzes the reversible phosphorylation of dTMP to deoxythymidine diphosphate (dTDP), which is an important step for cellular DNA synthesis (Cassera et al., 2011; Krungkrai and Krungkrai, 2016; Vanoevelen et al., 2022). It has a broad substrate specificity, which distinguishes it from other homologs, making it a suitable target (Reyes et al., 1982; Cassera et al., 2011; Krungkrai and Krungkrai, 2016). *P. falciparum* thymidylate monophosphate kinase (*Pf*TMPK) is involved in the pyrimidine biosynthetic pathway, a *de novo* pathway responsible for the production of pyrimidines, which are necessary for rapid DNA replication (Reyes et al., 1982; Cassera et al., 2011; Krungkrai and Krungkrai, 2016). The pathway is preferred because the parasite is highly dependent on it since it lacks pyrimidine salvage enzymes (Cassera et al., 2011; Krungkrai and Krungkrai, 2016).

So far, thiourea has been shown to exhibit inhibitory activity against *Pf*TMPK, the compound that was first discovered to inhibit the TMPK of *Mycobacterium tuberculosis* (*Mt*TMPK) (Cui et al., 2012; Patrick and Turner, 2020). The inhibitory effect of thiourea on *Pf*TMPK is however weaker than that of *Mt*TMPK. Several analogs containing thiourea or urea have since been tested for antimalarial activity through the inhibition of *Pf*TMPK (Cui et al., 2012). However, the analogs have mostly exhibited weak inhibition against *Pf*TMPK. The most active analog, phenylurea, exhibited an EC_50_ of 28 nM but was reported to be a weak inhibitor of *Pf*TMPK with a binding affinity of 200 µM (Cui et al., 2012; Chen et al., 2018). It was also observed that ureas showed more antimalarial activities than thioureas (Cui et al., 2012; Patrick and Turner, 2020).

In this study, new antimalarials are predicted for *P. falciparum* by virtually screening an African natural product library against *Pf*TMPK. Recently, the development of quantitative structure–activity relationship (QSAR) models has contributed significantly to antimalarial discovery (Neves et al., 2020). As such, this study explores the structural insights for *Pf*TMPK inhibition using QSAR modeling, pharmacophore mapping, and docking studies (Ojha and Roy, 2013) to select the most plausible antimalarial lead compounds with good pharmacological profiles from compounds in the AfroDB database. Molecular dynamic simulations were also undertaken to provide insights into the binding mechanisms of *Pf*TMPK.

## Methodology

### *In-Silico* Absorption, Distribution, Metabolism, Excretion, and Toxicity Filtering of the Natural Product Database

A total of 885 African natural products were retrieved from AfroDB (Ntie-Kang et al., 2013) in “.sdf” format for absorption, distribution, metabolism, excretion, and toxicity (ADMET) analysis. The compounds were screened *via* FAF-Drugs4 (Lagorce et al., 2017) to elucidate their pharmacokinetic, structural, and physicochemical properties. The physicochemical filter used for the compounds was “Drug likeness”. The compounds were categorized as “rejected”, “intermediate”, or “accepted” by FAF-Drugs4. “Accepted” compounds were then chosen for molecular docking.

### Virtual Screening

The “accepted” compounds from the FAF-Drugs4 ADMETox prediction were docked against *Pf*TMPK, using AutoDock Vina (Trott and Olson, 2010) integrated with PyRx (Dallakyan and Olson, 2015). The substrate of *Pf*TMPK (TMP) was also extracted from the complex (Protein Data Bank (PDB) ID: 2wwf) and added to the library of “accepted” compounds for docking. Before the docking, the compounds were energy minimized with OpenBabel using the universal force field (uff) before converting to “.pdbqt” formats (Kwofie et al., 2021). A grid box size of 17.3, 9.7, and 11.2 Å and center dimensions of 42.5, 46.7, and 47.6 Å all in the x, y, and z coordinate axes were used, respectively.

### Docking Method Validation

For validation of the performance of the docking technique, the SMILES of five potent inhibitors (compounds 28, 30, 53, 54, and 55) against *Pf*TMPK (Cui et al., 2012) were used to generate their decoys *via* RApid DEcoy Retriever (RADER) (Wang et al., 2017). A total of 243 decoys were obtained and screened together with the five inhibitors against *Pf*TMPK using AutoDock Vina. The docking results were used to generate a receiver operating characteristic (ROC) curve, and the area under the curve (AUC) was computed using easy ROC Ver. 1.3 (Goksuluk et al., 2016). Parameters used for the ROC generation and calculation of AUC were a non-parametric method for curve fitting (DeLong et al., 1988) for SE estimation and CI as well as a Type I error of 0.05.

The co-crystallized ligands of *Pf*TMPK were removed from their binding site and re-docked against *Pf*TMPK. The best-predicted docking pose of each ligand was superimposed with its respective experimental co-crystallized ligand pose, and their root mean square deviation (RMSD) was calculated using LigAlign (Heifets and Lilien, 2010).

### Molecular Dynamic Simulations of Complexes

Molecular dynamics simulations were performed using the *Pf*TMPK in complex with TMP and the respective chosen potential lead compounds. GROningen MAchine for Chemical Simulation (GROMACS) version 5.1.4 (Van Der Spoel et al., 2005) was used to perform the molecular dynamics simulations using the GROMOS96 43A1 force field. The topology of the compounds was generated using PRODRG (Schüttelkopf and Van Aalten, 2004). As part of the preparation before the simulation, the complexes were first solvated in a 1-nm dodecahedron water box and neutralized by adding one positive ion. The complexes were relaxed through energy minimization to remove any steric clashes or bad geometry and equilibrated to the required temperature (300K) and density (1,020 kg/m^3^). After the respective systems were equilibrated and set in the desired temperature and density, a 100-ns production run was performed, and the results of the simulations were analyzed using Xmgrace version 5.1.25 (Vaught, 1996).

### *In-Vitro* Parasite Growth Inhibition Assay

One of the potential lead compounds, aurantiamide acetate, was tested for anti-plasmodial activity using the SYBR Green I fluorescence assay as described previously (Smilkstein et al., 2004). Stock concentrations of 100 mM [100% dimethyl sulfoxide (DMSO)] of compounds were diluted with culture media to a working concentration of 100 µM (0.1% DMSO). A serial dilution of 1:2 concentrations of the compound (10 to 0.781 µM) was prepared for the assay. Artesunate was the reference drug diluted from 100 to 3.125 nM. Test wells were initially seeded with 90 µl of ring-stage (synchronized) parasitized red blood cells (pRBCs), culture media, and pRBCs at 2% hematocrit and 1% parasitemia. An aliquot of 10 µl of each concentration was dispensed into each well in triplicates. The wells containing RBCs (2% hematocrit), pRBCs, and cultured protoplast washing (CPW) media served as negative and blank controls and were then incubated for 48 h. An aliquot of 100 µl of 4× buffered SYBR Green I (0.20 µl of 10,000× SYBR Green I/ml of 1× phosphate-buffered saline) was then added for a further 30 min at 37°C. The presence and amount of pRBCs were detected by fluorescence using the Guava EasyCyte HT FACS machine (Millipore, Billerica, MA, USA), and parasitemia was recorded in percentages. The IC_50_ was extrapolated from non-linear regression curves of percentage inhibition versus log-concentration curves from GraphPad Prism (Graph Pad Software, San Diego, CA, USA) using algorithms obtained from flow cytometry (fluorescence-activated cell sorting (FACS)) data. The experiment was repeated using an asynchronized parasite culture using the same protocol.

## Results and Discussion

### Target Structure

**Figure 1:**
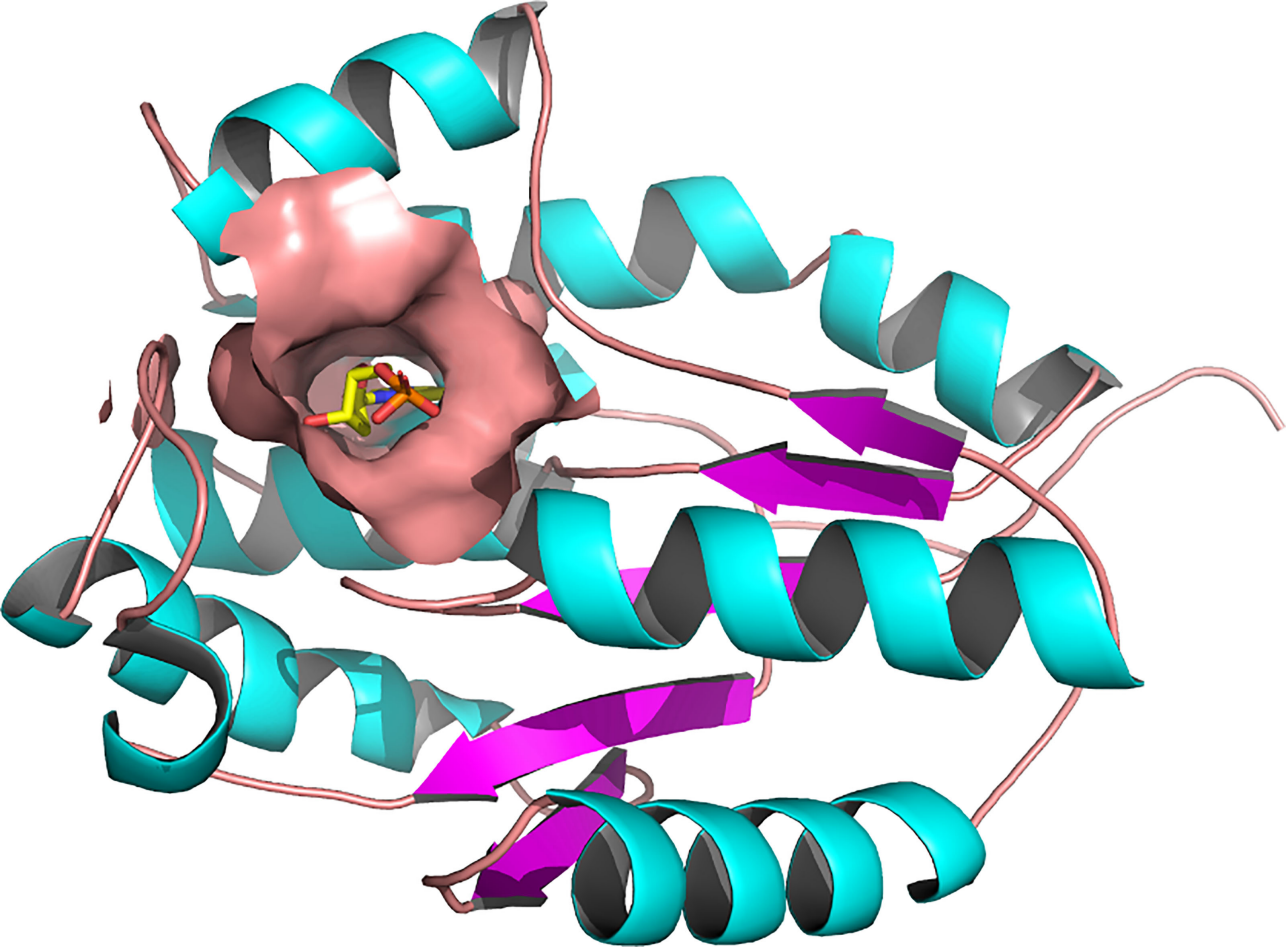
Three-dimensional structure of *Plasmodium falciparum* thymidylate monophosphate kinase (PDB ID: 2WWF) and its active site (Whittingham et al., 2010). A cartoon representation of the 3D structure of *Pf*TMPK. The surface representation shows the active site of *Pf*TMPK and binding in its natural substrate, thymidine monophosphate (TMP), represented by yellow sticks. Active site residues include Asp17, Lys21, Leu59, Phe44, Arg47, Pro45, Phe74, Arg78, Arg99, Tyr100, Ser103, Gly104, Tyr107, and Tyr153 (Kandeel et al., 2009). The protein structure was retrieved from the Protein Data Bank, and image was generated with PyMOL.

The three-dimensional structure of *Pf*TMPK retrieved from the PDB (PDB ID: 2wwf) was solved using X-ray crystallography at a resolution of 1.89 Å (Whittingham et al., 2010). The 2wwf is a homo-oligomer (homodimer) consisting of three chains of *Pf*TMPK in complex adenosine diphosphate (ADP) and thymidine monophosphate (TMP), which is a natural substrate. Other ligands like sodium-ion and glycerol are also present. **Figure 1** shows chain A of 2wwf with the active site, and TMP is highlighted. The protein has a sequence length of 212 and is significantly different from the human TMPK with a sequence identity of 36.9%.

### Absorption, Distribution, Metabolism, Excretion, and Toxicity-Acceptable Compounds

ADMET tests are performed to eliminate compounds that may be weak drug candidates so that potential drug-like compounds are prioritized (Sliwoski et al., 2013). FAF drug server uses quantitative QSAR models to predict specific properties or toxicological endpoints of compounds (Lagorce et al., 2017). The predicted properties were compared with the standard range of accepted values concerning the chosen physicochemical filter, which was “drug-likeness”. The filtered compounds were categorized as “rejected”, “intermediate”, or “accepted”. The “accepted” compounds are those with no structural alerts and satisfy the physicochemical filter. Intermediate and rejected compounds show some structural alerts and do not satisfy completely the physicochemical filter. Structural alerts are substructures that are related to mutagenic and carcinogenic properties, which are undesirable for drug-likeness (Benigni and Bossa, 2006). After the compounds were screened using the FAF drug, 91 compounds categorized as “accepted” were used for docking against *Pf*TMPK.

### Docking Protocol Validation

**Figure 2:**
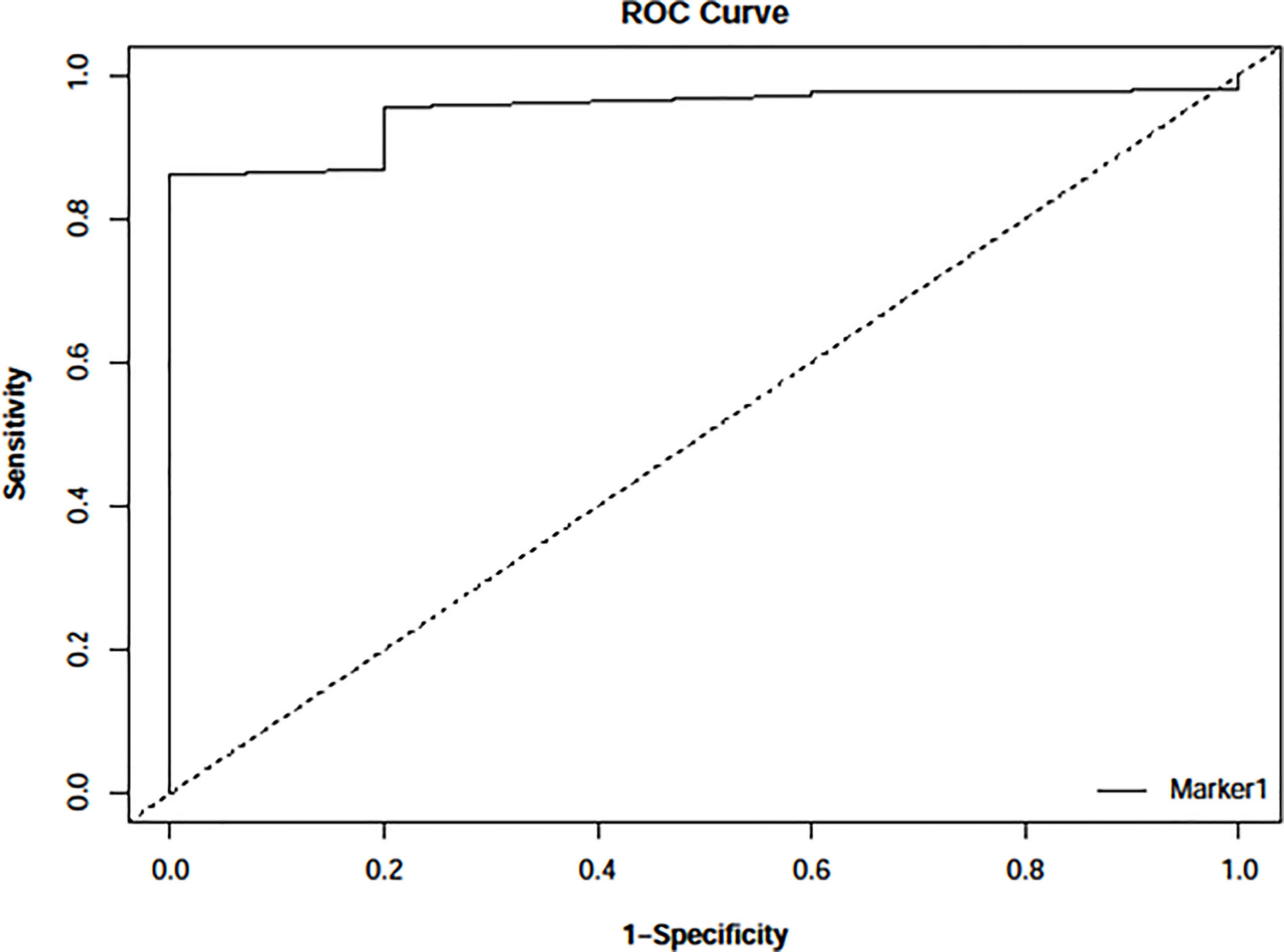
Receiver operating characteristic (ROC) curve generated with easyROC (Goksuluk et al., 2016) after docking 243 decoys and 5 potent inhibitors against *Pf*TMPK using AutoDock Vina *via* PyRx version 0.8. The area under the curve (AUC) obtained was 0.94897 with a p-value of 9.776159e−78.

To validate AutoDock Vina’s ability to distinguish between active and inactive compounds concerning *Pf*TMPK, a ROC curve (**Figure 2**) was generated after virtually screening five potent inhibitors of *Pf*TMPK (Cui et al., 2012) (**Table 1**) and their decoys against the receptor. The AUC value was calculated to assess the docking performance. AUC value less than 0.5 is considered a poor discrimination ability, from 0.5 to 0.7 is considered moderate, and greater than 0.7 is acceptable (Mandrekar, 2010). AUC value very close to 1 indicates an excellent discriminatory ability of the docking model for the receptor (Mandrekar, 2010). The AUC obtained was 0.95 with a p-value of 9.776159e−78, indicating an excellent discriminatory ability of AutoDock Vina to distinguish between active compounds and decoys of *Pf*TMPK.

**Table 1:** List of potent inhibitors and co-crystallized ligands showing the Ki values (Cui et al., 2012), the respective PDB IDs, and RMSD values of the aligned co-crystallized and re-docked ligands.

Compound	Type	K_i_ (µM)	Ligand	PDB ID	RMSD (Å)
Thymidine monophosphate	Substrate	(K_m_ = 11 µM)	TMP	2wwf	0.376
Compound 28	Inhibitor	31	74W	2yof (chain C)	1.768
Compound 30	Inhibitor	11	74X	2yog	1.741
Compound 53	Inhibitor	25	WMJ	2yoh	1.073
Compound 54	Inhibitor	27	–	–	–
Compound 55	Inhibitor	11	–	–	–

PDB, Protein Data Bank; RMSD, root mean square deviation.

To validate further the docking approach used, four co-crystallized *Pf*TMPK ligands (three of which are complexed with inhibitors and a substrate) were retrieved from their PDB crystal structures and re-docked against the *Pf*TMPK protein structures. The co-crystallized binding poses of the ligands were superimposed to their respective predicted docking poses, and RMSDs less than 2 Å were obtained for all of them. This suggests that AutoDock Vina reasonably distinguished between active and inactive compounds for *Pf*TMPK and accurately predicted binding poses.

### Virtual Screening Analysis and Lead Identification

Ninety-one pre-filtered drug-like compounds were screened against *Pf*TMPK together with its natural substrate and known potent inhibitors. After docking, thirteen of the drug-like compounds had binding affinities greater than the protein’s substrate and were selected as hits (**Table 2**). The binding energies of three potent inhibitors were −8.9, −9.3, and −9.7 kcal/mol for compounds 28, 53, and 55, respectively, which fell within the same range as the hits. The lowest energies among them were −10.5 and −10.2 kcal/mol for compounds 54 and 30, respectively, which were not far from the lowest among the hits (−9.9 kcal/mol). This is a good indication of the potential antimalarial activity of the hits. Lead compounds were then selected among the hits. Four structural properties of a molecule that ensure proper binding at the active site and inhibitory effect against *Pf*TMPK, developed using QSAR analysis, pharmacophore modeling, and docking studies with a set of thymidine analogs that have well-defined *Pf*TMPK inhibitory activity, were considered in selecting possible lead compounds (Ojha and Roy, 2013). These properties are the presence of –NH fragment, –OH group, urea moiety, and a considerable amount of oxygen atoms (Ojha and Roy, 2013).

**Table 2:** List of hits showing their structures, binding energies, and structural properties.

Compound	Binding energy (kcal/mol)	Structural properties			
		NH fragment	OH group	More O-atoms	Urea moiety
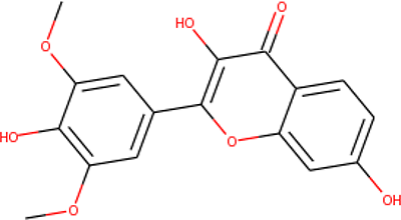 ZINC14644461	−9.9	✖	✔	✔	✖
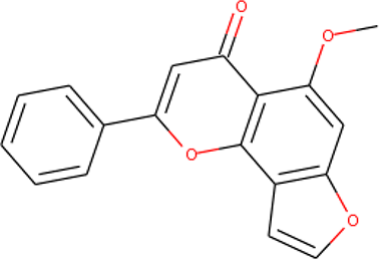 ZINC14677166	−9.7	✖	✖	✔	✖
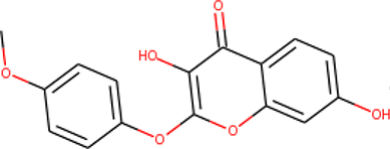 ZINC95486297	−9.6	✖	✖	✔	✖
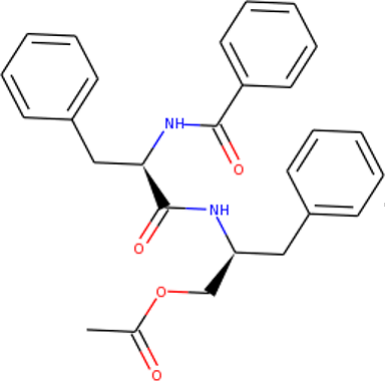 ZINC13374323	−9.4	✔	✖	✔	✔
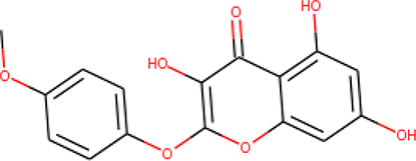 ZINC95486293	−9.4	✖	✔	✔	✖
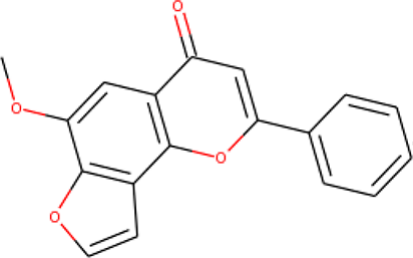 ZINC14504006	−9.3	✖	✖	✔	✖
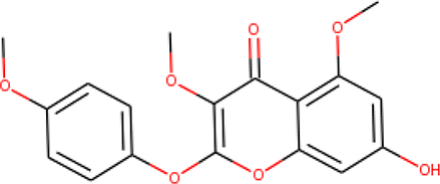 ZINC95486295	−9.2	✖	✔	✔	✖
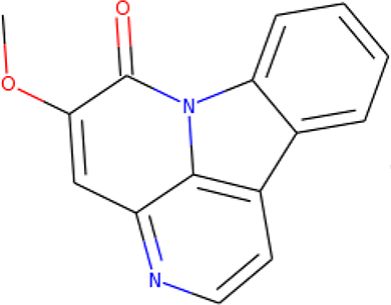 ZINC13282986	−9	✖	✖	✖	✖
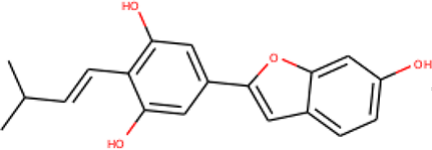 ZINC87493012	−9	✖	✔	✔	✖
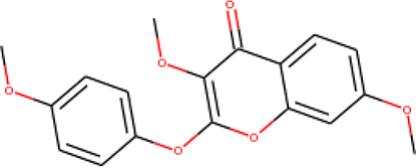 ZINC95486296	−9	✖	✔	✖	✖
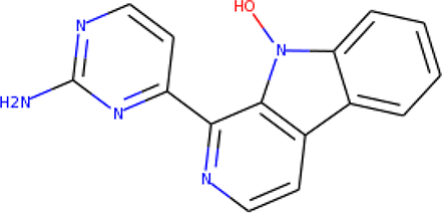 ZINC13365918	−8.9	✔	✖	✔	✔
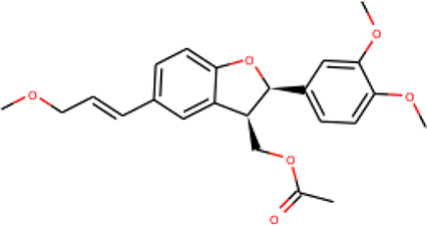 ZINC95486004	−8.9	✖	✖	✔	✖
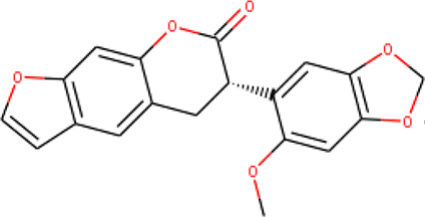 ZINC05357841	−8.8	✖	✖	✔	✖
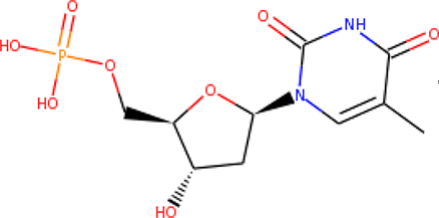 TMP	−8.7	—	—	—	—
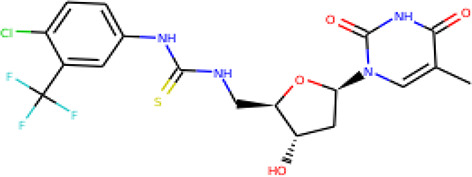 Compound 28	−9.3	—	—	—	—
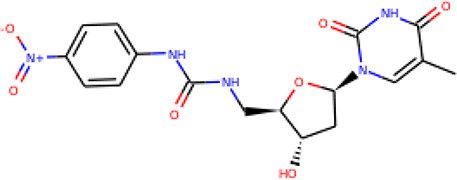 Compound 30	−10.2	—	—	—	—
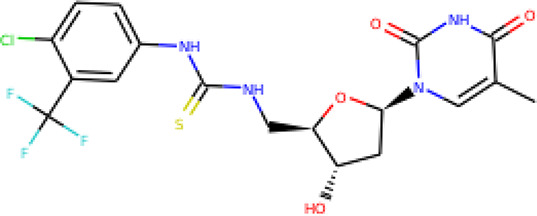 Compound 53	−8.9	—	—	—	—
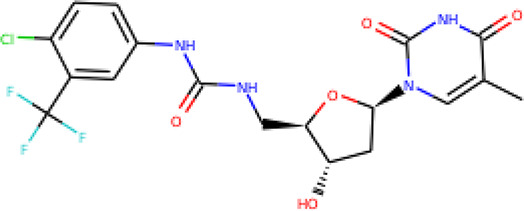 Compound 54	−10.5	—	—	—	—
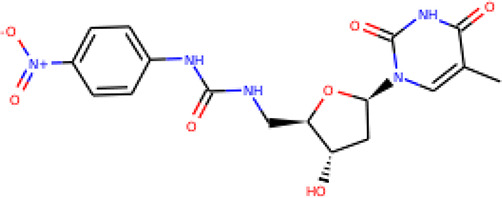 Compound 55	−9.7	—	—	—	—

✔ is shown when the structural property applies to the compound, while ✖ is shown when the property does not apply. Structures and binding energies of the substrate (TMP) and the five potent inhibitors are included.

The molecular structures of the hit compounds were analyzed to identify any of the aforementioned structural properties. ZINC13374323 possesses –NH group, urea moiety, and a considerable amount of oxygen atoms, while ZINC13365918 also has all the properties but lacks oxygen atoms. ZINC14644461, ZINC95486293, ZINC95486295, and ZINC87493012 all have –OH groups and considerable amounts of oxygen atoms, while ZINC13282986 possesses none of the structural properties. The rest of the six compounds only possess oxygen atoms among the aforementioned structural properties. Most of the compounds seem to possess considerable amounts of O-atoms, which is an essential requirement for high binding affinity toward *Pf*TMPK. The most distinctive structural properties were the presence of NH fragment and urea moiety, which were exhibited only by ZINC13374323 and ZINC13365918. Although all four properties are essential for good inhibitory activity against *Pf*TMPK (Ojha and Roy, 2013), a ligand with at least three of the properties, placing priority on the distinctive properties (presence of -NH fragment and urea moiety), may exhibit a good inhibitory effect against the compound with no or little modifications. As such, ZINC13374323 and ZINC13365918 were considered the potential lead compounds among the 13 hits.

### Protein–Ligand Interactions

**Figure 3:**
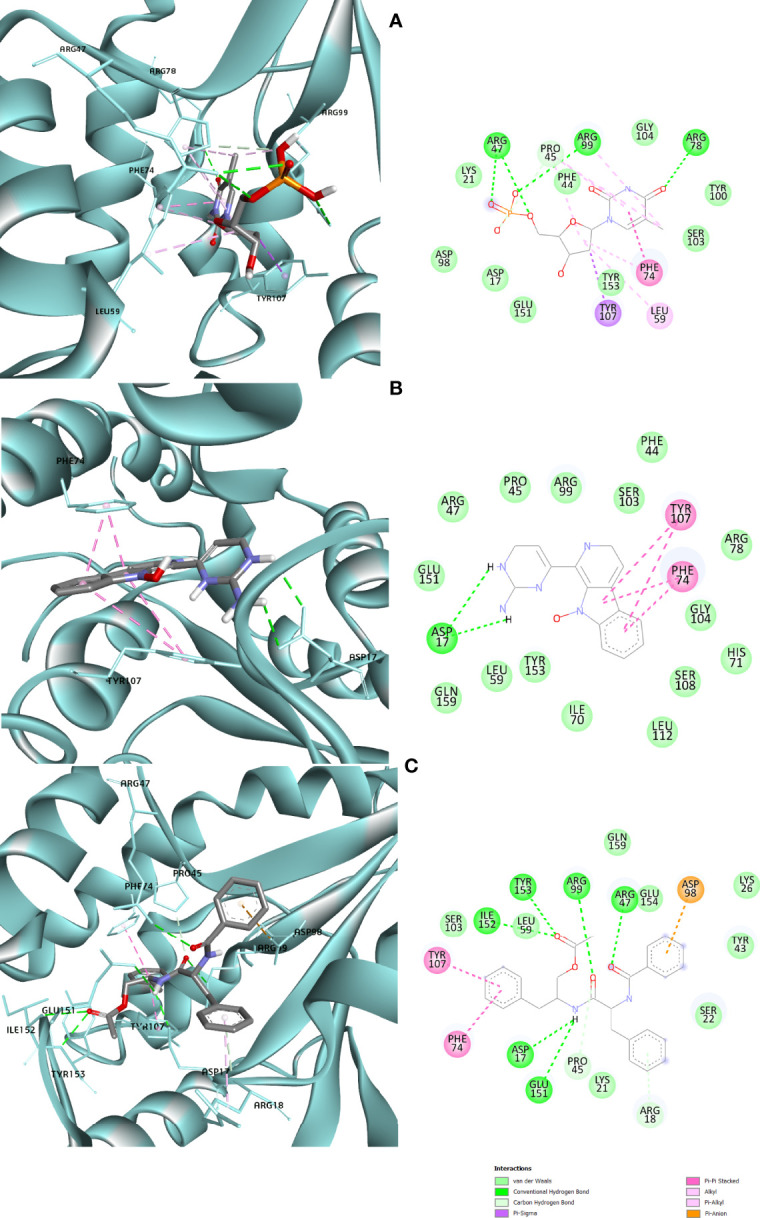
3D and 2D images of the protein–ligand interactions between *Pf*TMPK and **(A)** TMP, **(B)** ZINC13365918, and **(C)** ZINC13374323. TMP forms H-bonds with Arg47, Arg99, and Arg78; ZINC13374323 is shown to have H-bond interactions with Arg99, Arg47, Asp17, Glu151, Ile152, and Tyr153; ZINC13365918 forms H-bonds with only Asp17.

Protein–ligand molecular interactions of ZINC13374323 and ZINC13365918 were further studied and compared to the protein–ligand molecular interaction of compound 25, a *Pf*TMPK inhibitor thymidine analog, which has been shown to exhibit very high activity against the protein (Ojha and Roy, 2013). Compound 25 has been reported to share molecular interactions with protein residues Arg78, Arg99, Arg47, Asp17, Ser22, Phe74, and Tyr43 (Ojha and Roy, 2013). In **Figure 3A**, we show the substrate to share interactions with similar residues such as Arg78, Arg99, Arg47, and Phe74. From **Figure 3C**, ZINC13374323 is also shown to have interactions with common residues such as compound 25. ZINC13365918, on the other hand, exhibited interactions with Asp17 and Phe74 among the listed residues (**Figure 3B**). This warranted the experimental characterization of ZINC13374323 to corroborate potential inhibitory activity against *Pf*TMPK. H-bond interactions with Asp17 and Pi-Pi stacked interactions with Tyr107 and Phe74 are common among both ligands. This may indicate the importance of the residues for effective binding. One can suggest that the lack of more O-atoms in ZINC13365918 significantly affects its ability to form H-bonds with important residues such as Arg47 and Arg99. TMP and ZINC13374323 were able to form H-bonds with both Arg47 and Arg99 using their O-atoms. However, a lack of an OH-group did not seem to affect the ligands’ interactions, since none of them formed any bonds with it.

### Molecular Dynamics Simulations

#### Stability of Protein–Ligand Complexes

**Figure 4:**
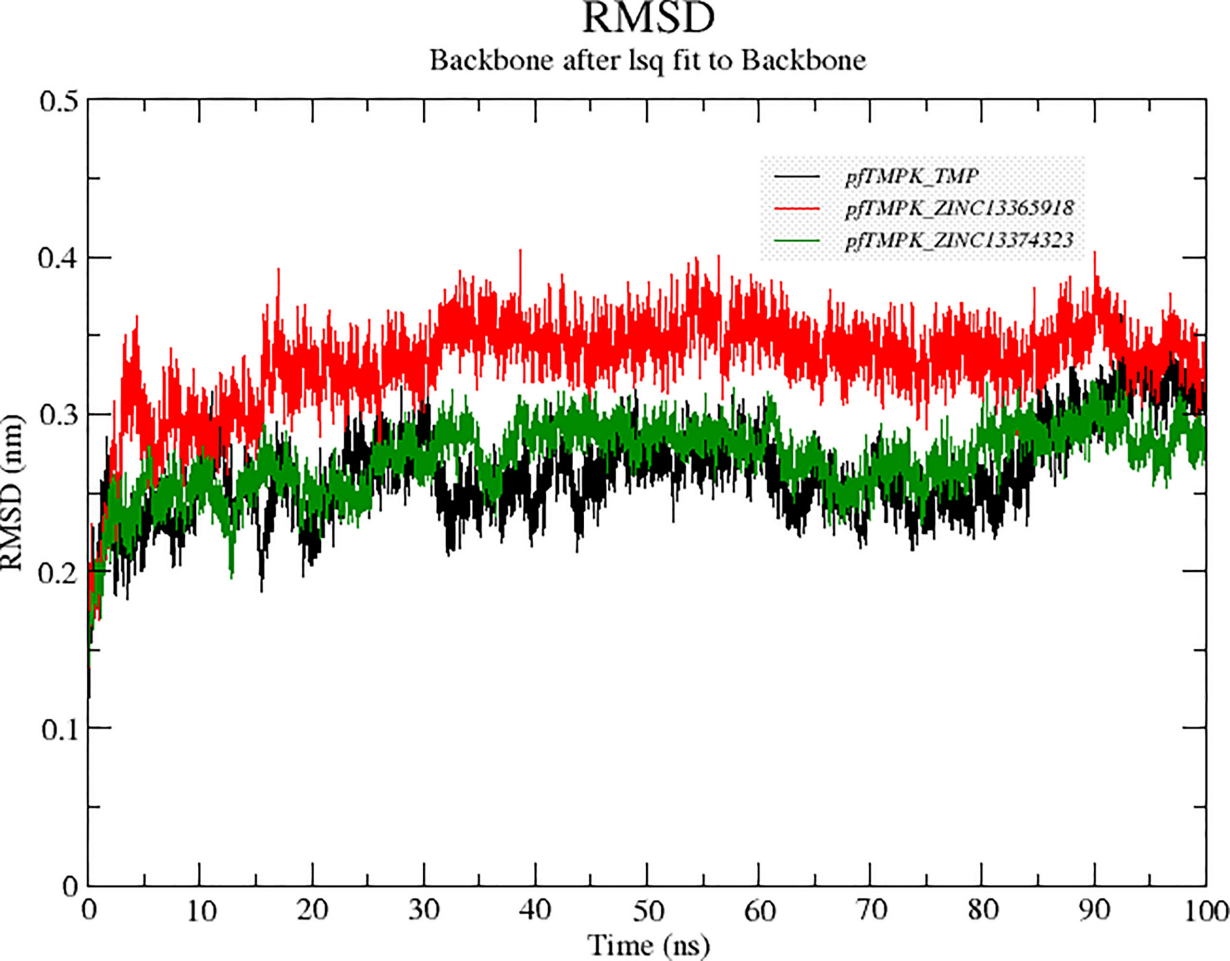
Root mean square deviation (RMSD) graphs comparing the RMSDs of *Pf*TMPK in complex with its natural substrate (TMP) and the respective lead compounds. The RMSD graph for *Pf*TMPK–TMP is colored black, for *Pf*TMPK–ZINC13365918 red, and for *Pf*TMPK–ZINC13374323 green.

Molecular dynamics simulations were performed to compare the stability of the distinctive protein–ligand complexes of *Pf*TMPK with TMP, ZINC13374323, and ZINC13365918. To accomplish that, the RMSDs of the complexes were generated after the simulation (**Figure 4**). The results showed that *Pf*TMPK–ZINC13374323 behaved very similarly to *Pf*TMPK–TMP in terms of stability. Complex *Pf*TMPK–ZINC13365918 shows a higher RMSD but has much fewer fluctuations than *Pf*TMPK–TMP and *Pf*TMPK–ZINC13374323, maintaining an RMSD of 0.34 nm from 15- to 90-ns simulation time. Complexes *Pf*TMPK–TMP and *Pf*TMPK–ZINC13374323 exhibited RMSD from 0.25 nm at the first 20 ns of the simulation to 0.26 nm between 20 ns and 60 ns and 0.27 nm in the final 10 ns of the simulation.

#### Influence of Ligand Binding on the Flexibility of *Pf*TMPK

**Figure 5:**
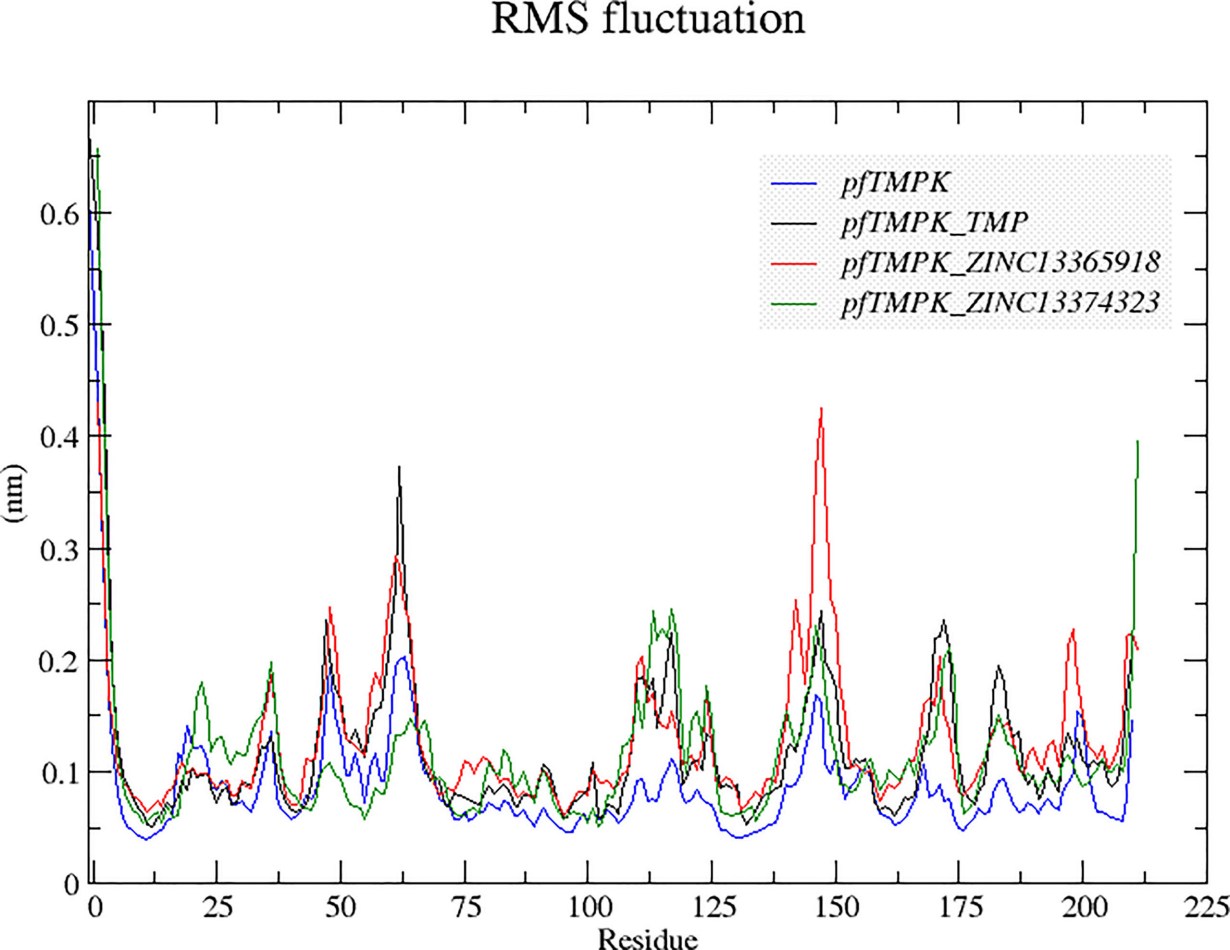
Root mean square fluctuation (RMSF) graphs comparing the behaviors of the residues of *Pf*TMPK in complex with its natural substrate (TMP) and the respective compounds with those of the unbound protein. The RMSF graph for the unbound *Pf*TMPK is shown in blue, for *Pf*TMPK–TMP black, for *Pf*TMPK–ZINC13365918 red, and green for *Pf*TMPK–ZINC13374323.

A comparison of the behavior of the protein residues in each complex was also done using the root mean square fluctuations (RMSFs) (**Figure 5**). It can be observed from the graph that the protein residues experienced higher fluctuations in *Pf*TMPK–ZINC13365918 than in the other complexes and the unbounded *Pf*TMPK. The fluctuations of *Pf*TMPK–ZINC13374323 and *Pf*TMPK–TMP seem to be very close, indicating a similar margin of flexibility within the protein when complexed with either ZINC13374323 or TMP. A similar observation was made with their RMSDs. However, all the complexes generally experienced more fluctuations than the unbound protein throughout the simulation. The fluctuations were most probably induced by the effect of the binding of the ligands.

#### Binding Interactions During Molecular Dynamics Simulations

**Figure 6:**
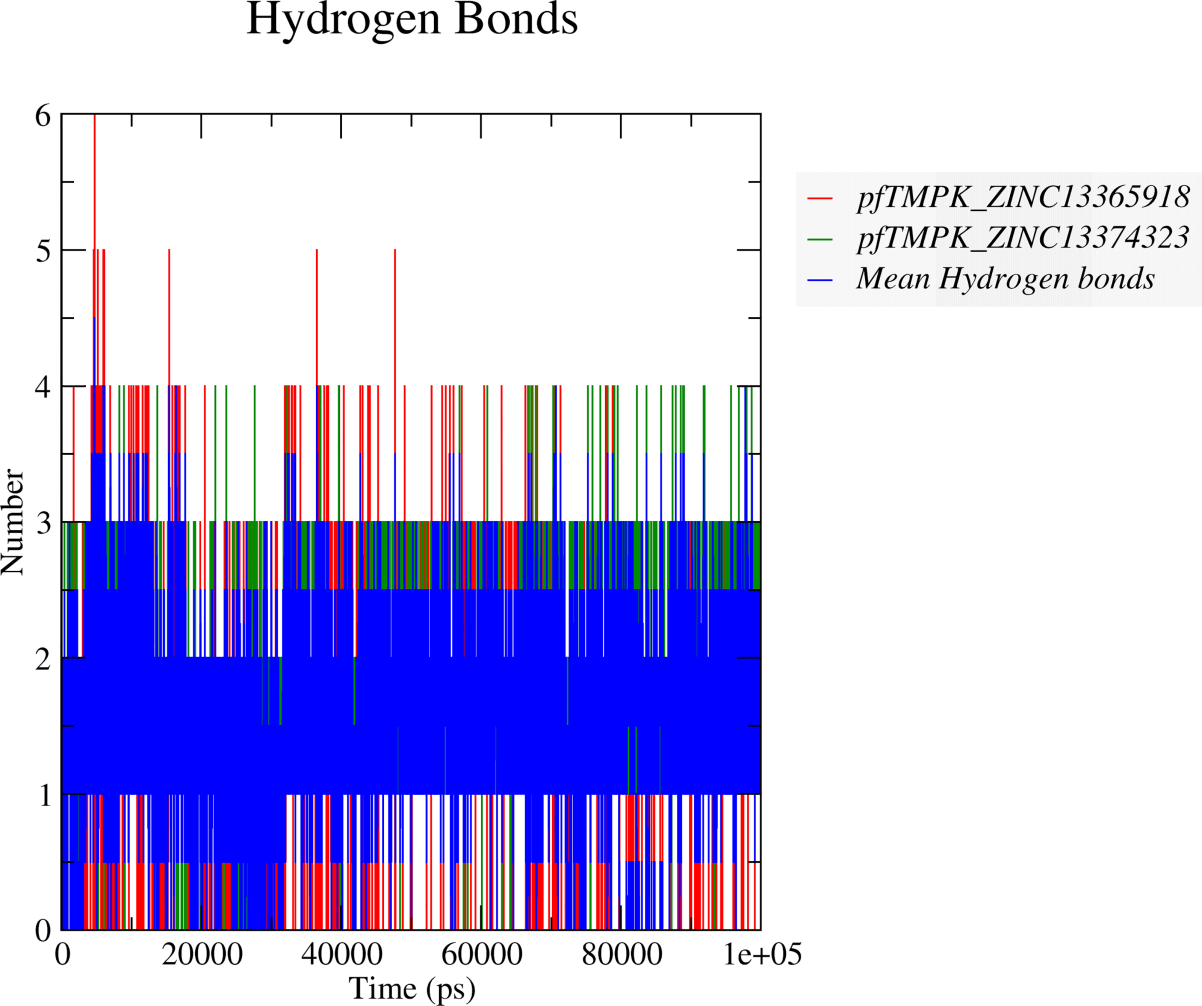
Hydrogen bond predictions between the complexes of *Pf*TMPK and the respective potential lead compounds. The hydrogen bonds formed between *Pf*TMPK and ZINC13365918 are shown as red lines, while those of the protein and ZINC13374323 are shown as green lines. The mean hydrogen bonds formed throughout the simulation in both complexes are highlighted in blue.

To determine if ZINC13374323 and ZINC13365918 can maintain strong interactions with *Pf*TMPK, the numbers of hydrogen bonds formed between the respective compounds and *Pf*TMPK throughout the simulation were analyzed. This suggests a continuous bind of the ligand in the binding site under harsh dynamic conditions. The average number of hydrogen bonds (**Figure 6**) shared between *Pf*TMPK–ZINC13365918 and *Pf*TMPK–ZINC13374323 complexes during the entire simulation decreased to between 1 and 2. Complex *Pf*TMPK–ZINC13374323 formed 3 and 4 hydrogen bonds more frequently during the simulation as compared to *Pf*TMPK–ZINC13365918. Complex *Pf*TMPK–ZINC13365918 reached as high as 5 and 6 hydrogen bonds, but this happened very few times in the simulation. The average hydrogen bonds shown were consistent with the H-bond interactions obtained after molecular docking (**Figures 3B, C**).

### Exploring Biological Activities of Potential Lead Compounds

The common name of ZINC13374323 is aurantiamide acetate, which has been shown to exhibit anti-inflammatory and antiviral activities in influenza-infected cells (Zhou et al., 2017). The compound is a constituent of herbs such as the bark of *Albizia adianthifolia* and *Brillantaisia lamium*, where it has been shown to exhibit antimicrobial activity (Tamokou et al., 2011; Tamokou et al., 2012). Interestingly, aurantiamide acetate is also an ingredient of *A. annua*, the Chinese herb from which artemisinin was discovered (Milne et al., 2018). ZINC13365918 is a pyrimidine analog with the name *N*-hydroxyannomontine. *N*-Hydroxyannomontine has been shown to exhibit antileishmanial activity against *Leishmania braziliensis* and *Leishmania guyanensis* (Costa et al., 2006). The toxicity profiles of both compounds as shown in **Table 3** indicate the compounds to be generally safe, with aurantiamide acetate tending to cause some irritation. The toxicity profile was generated with OSIRIS DataWarrior version 5.5.0 (Sander et al., 2015).

**Table 3:** Toxicity profile of potential lead compounds.

	Mutagenicity	Tumorigenic	Reproductive effectiveness	Irritant
**ZINC13374323** **(aurantiamide acetate)**	None	None	None	High
**ZINC13365918** **(*N*-hydroxyannomontine)**	None	None	None	None

**Figure 7:**
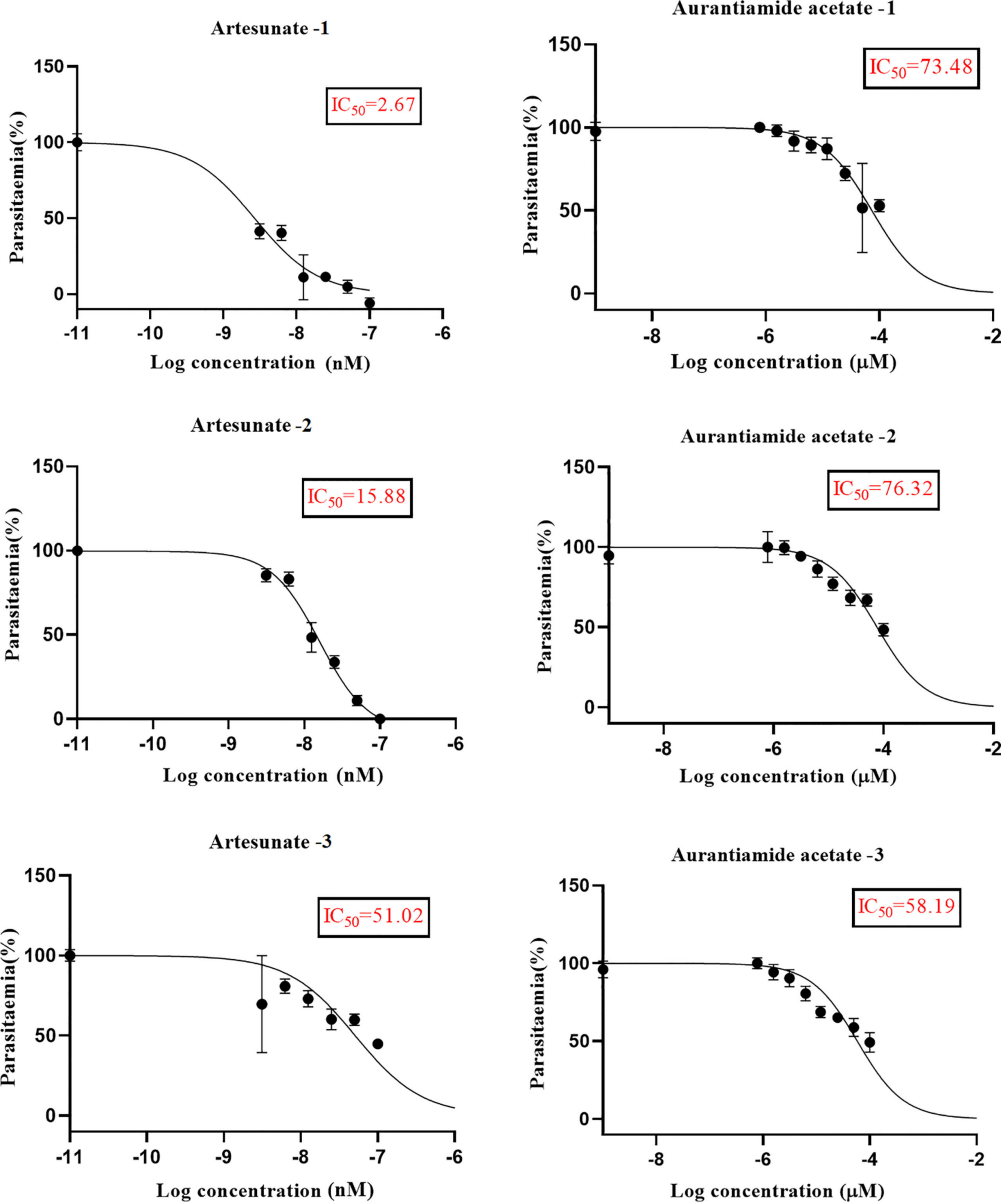
The inhibition curves of artesunate and aurantiamide acetate for the synchronized culture of *Plasmodium falciparum* 3D7 ring stages.

We explored the anti-plasmodial activity of aurantiamide acetate further since it is commercially available. *N*-Hydroxyannomontine is currently not commercially available; as such, *in vitro* experimentation for this analog was not carried out. An *in vitro* parasite growth inhibition assay was prepared for the *P. falciparum* 3D7 strain using the SYBR Green I fluorescence assay (Smilkstein et al., 2004). Artesunate was used as the positive control. The compounds were added to synchronized cultures of the ring stages, and inhibition of the parasite’s growth was determined through IC_50_, with the experiment conducted in triplicates. The IC_50_ values obtained for artesunate were comparable to those reported previously with the average being 23.22 nM (Touré et al., 2008; Quashie et al., 2013). The average IC_50_ value obtained for aurantiamide acetate was 69.33 μM (**Table 4**). The inhibition curves of both compounds are shown in **Figure 7**. A single screen of aurantiamide acetate was also performed on asynchronized cultures of *P. falciparum* 3D7 strain. The IC_50_ value achieved for the asynchronized culture was >100 μM (**Supplementary Figure 1**), which indicates an increase in the potency of the compound when screened against the ring stages of the parasite. This could imply a clue into the target specificity of the compound but requires further investigation; therefore, a stage-of-action study to assess the potency of the compound against different asexual stages is recommended. The activity of aurantiamide acetate, despite being appreciable for a natural compound, must be further improved through optimization to cater to its lack of O-atoms, which is necessary for *Pf*TMPK inhibition.

**Table 4:** IC_50_ values of artesunate and aurantiamide acetate for the synchronized culture of *Plasmodium falciparum* 3D7 ring stages.

	Artesunate (nM)	Aurantiamide acetate (μM)
IC_50_—Experiment 1	2.76	73.48
IC_50_—Experiment 2	15.88	76.32
IC_50_—Experiment 3	51.02	58.19
Mean IC_50_	23.22	69.33
SD	24.95	9.751
SEM	14.41	5.630

## Significance of the Study

In recent years, a minimal number of natural products have made it to antimalarial lead optimization projects (Guantai and Chibale, 2011). Natural products have proven to be a rich source of antimalarials throughout the history of malaria drug discovery, with breakthroughs like quinine and artemisinin being prime examples (Saxena et al., 2003; Itokawa et al., 2008). Most other drugs like clindamycin and azithromycin have also had their foundation in natural products (Guantai and Chibale, 2011). This shows that developing new drug candidates from natural product sources remains a favorable line of research for antimalarial drug development. It is evident, however, that most compounds developed from natural products show moderate inhibition activity (Saxena et al., 2003; Guantai and Chibale, 2011). As such, through the incorporation of *in silico* techniques, we can focus on compounds with desirable properties with the potential to become lead compounds and later become drug candidates through further optimization. The thirteen hits obtained after molecular docking represent compounds that can compete with the natural substrate of *Pf*TMPK based on their binding affinity. By screening those compounds against important structural properties obtained through extensive *in silico* work (Ojha and Roy, 2013), we have been able to highlight two natural products, namely, aurantiamide acetate and *N*-hydroxyannomontine, as potential lead compounds. The binding affinities of the compounds as compared to known active inhibitors of *Pf*TMPK corroborate this claim. Also, further experimental *in vitro* studies of the biological activity of aurantiamide acetate against the *P. falciparum* 3D7 strain show the compound to express appreciable antimalarial activity (IC_50_ of 69.33). *In vitro* experimentation could not be done for *N*-hydroxyannomontine because it is not commercially available. It will be necessary to synthesize and test the compound in future studies and also develop cytotoxicity assays for both compounds. To develop these compounds into lead compounds, it is crucial to optimize them, taking into account the structural properties needed for effective binding as earlier described (Ojha and Roy, 2013).

Resistance to chloroquine, a cheap and efficacious antimalarial drug, has led to the change to artemisinin-based combination therapy (ACT) as the recommended first-line treatment option. Chloroquine resistance was first observed in Southeast Asia (in Thailand in 1957), which spread globally (Packard, 2014) and rendered chloroquine useless. However, resistance to ACT has been detected again in Southeast Asia with hints of delayed parasite clearance in Africa (Global Malaria Program, 2015). Delayed parasite clearance, however, may not necessarily lead to treatment failure, but in the Greater Mekong Subregion, treatment failures following treatment with an ACT have only been observed where there is resistance to the partner drug (World Health Organization, 2018). The drugs used in the ACT are few, necessitating the need to have additional compounds, thus the significance of our finding of aurantiamide acetate as a potential antimalarial compound. Moreover, it is known to possess anti-inflammatory properties (Zhou et al., 2017), which is good. Although its anti-plasmodial activity *in vitro* is not comparable to that of artesunate, we believe that the addition of oxygen atoms to the molecule will significantly increase its performance. Studies have reported that dried leaves of *A. annua* used as a tea infusion in treating malaria have a higher potency than pure artemisinin (Weathers, 2014). Using the dried-leaf treatment has also been shown to overcome resistance to artemisinin. These effects may be due to the other chemical components of *A. annua*, which enhance the bioavailability and efficacy of artemisinin even though they have significantly less potent anti-plasmodial activity than artemisinin (Weathers, 2014). Since aurantiamide acetate is a constituent of *A. annua*, we recommend further studies to determine its ability to reduce resistance to artemisinin and/or enhance anti-plasmodial activity when used as a partner drug in ACT.

## Conclusion

A total of 885 compounds retrieved from AfroDB were screened in FAF-Drugs4 server, which produced 91 ADMET-acceptable compounds. Thirteen compounds out of the 91, which scored higher binding energy than the substrate of *Pf*TMPK, were selected as hits. Four structural properties of a molecule that ensures proper binding at the active site and inhibitory effect against *Pf*TMPK were considered in selecting lead compounds. ZINC13374323 and ZINC13365918 were selected as plausible lead compounds since they exhibited three of the structural properties. ZINC13374323 shares interactions with many similar residues as a *Pf*TMPK inhibitor thymidine analog, which has been shown to exhibit high activity against the protein. ZINC13374323, also known as aurantiamide acetate, is an ingredient of *A. annua* and exhibits anti-inflammatory, antiviral, and antimicrobial activities. ZINC13365918, on the other hand, is a pyrimidine analog that has been shown to exhibit antileishmanial activity. Analysis of the molecular dynamics simulations of the lead compounds complexed with the protein showed the complex of *Pf*TMPK and ZINC13374323 to have similar RMSD and RMSF as that of the protein in complex with its natural substrate, TMP. *In vitro* testing of aurantiamide acetate for anti-plasmodial activity resulted in an IC_50_ of 69.33 μM. The compounds could be used as scaffolds for lead optimization.

## Data Availability Statement

The original contributions presented in the study are included in the article/**Supplementary Material**. Further inquiries can be directed to the corresponding author.

## Author Contributions

AG, MW, and SK developed the concept and designed the study. KE conducted the computational aspects of the study and drafted the manuscript. MT-T, AL, SN, and GD performed the *in vitro* experiments of the study. AG, MW, and SK edited and proofread the manuscript. All authors contributed to the manuscript.

## Conflict of Interest

The authors declare that the research was conducted in the absence of any commercial or financial relationships that could be construed as a potential conflict of interest.

## Publisher’s Note

All claims expressed in this article are solely those of the authors and do not necessarily represent those of their affiliated organizations, or those of the publisher, the editors and the reviewers. Any product that may be evaluated in this article, or claim that may be made by its manufacturer, is not guaranteed or endorsed by the publisher.
