# Supplementary material for: Targeting the Plasmodium falciparum’s Thymidylate Monophosphate Kinase for the Identification of Novel Antimalarial Natural Compounds
Source: Front Cell Infect Microbiol. 2022 May 25;12:868529. doi: 10.3389/fcimb.2022.868529 (PMC9174469; doi:10.3389/fcimb.2022.868529)
Supplement: Supplementary file 1 [file DataSheet_1.docx]

Supplementary Figure 1. **The inhibition curve of Aurantiamide acetate** for the asynchronized culture of *P. falciparum* 3D7.
